# Polycystin‐1 modulates RUNX2 activation and *osteocalcin* gene expression via ERK signalling in a human craniosynostosis cell model

**DOI:** 10.1111/jcmm.16391

**Published:** 2021-03-03

**Authors:** Maira Katsianou, Kostas A. Papavassiliou, Ilianna Zoi, Antonios N. Gargalionis, Dimitrios Panagopoulos, Marios S. Themistocleous, Christina Piperi, Athanasios G. Papavassiliou, Efthimia K. Basdra

**Affiliations:** ^1^ Department of Biological Chemistry Medical School National and Kapodistrian University of Athens Athens Greece; ^2^ Department of Neurosurgery Agia Sofia’ Children's Hospital Athens Greece

**Keywords:** craniosynostosis, dolichocephaly, ERK, mechanosensation, mechanotransduction, osteoblast differentiation, osteocalcin, PC1, RUNX2, trigonocephaly

## Abstract

Craniosynostosis refers to the premature fusion of one or more cranial sutures leading to skull shape deformities and brain growth restriction. Among the many factors that contribute to abnormal suture fusion, mechanical forces seem to play a major role. Nevertheless, the underlying mechanobiology‐related mechanisms of craniosynostosis still remain unknown. Understanding how aberrant mechanosensation and mechanotransduction drive premature suture fusion will offer important insights into the pathophysiology of craniosynostosis and result in the development of new therapies, which can be used to intervene at an early stage and prevent premature suture fusion. Herein, we provide evidence for the first time on the role of polycystin‐1 (PC1), a key protein in cellular mechanosensitivity, in craniosynostosis, using primary cranial suture cells isolated from patients with trigonocephaly and dolichocephaly, two common types of craniosynostosis. Initially, we showed that PC1 is expressed at the mRNA and protein level in both trigonocephaly and dolichocephaly cranial suture cells. Followingly, by utilizing an antibody against the mechanosensing extracellular N‐terminal domain of PC1, we demonstrated that PC1 regulates runt‐related transcription factor 2 (RUNX2) activation and *osteocalcin* gene expression via extracellular signal–regulated kinase (ERK) signalling in our human craniosynostosis cell model. Altogether, our study reveals a novel mechanotransduction signalling axis, PC1‐ERK‐RUNX2, which affects osteoblastic differentiation in cranial suture cells from trigonocephaly and dolichocephaly patients.

## 1 INTRODUCTION

Craniosynostosis refers to premature unsynchronized ossification and fusion of one or more cranial sutures during the perinatal stage. The condition presents in two forms: the syndromic and the non‐syndromic.^1^ The syndromic form is associated with other syndromes and genetic abnormalities (Apert, Crouzon, Pfeiffer, Muenke, Saethre‐Chotzen and Antley‐Bixler).^2^, ^3^ The isolated non‐syndromic form is characterized by fused sutures and represents about 75% of the reported cases.^4^, ^5^ According to the location of suture fusion, synostosis is divided into sagittal, metopic, bi‐ or unicoronal, and bi‐ or unilambdoid. The abnormal skull shape is characterized as dolichocephaly (most frequent form, 45%‐58% of all non‐syndromic craniosynostoses), trigonocephaly, brachycephaly, and anterior or posterior plagiocephaly.^6^, ^7^, ^8^, ^9^ Epidemiologically, it has been linked to various factors such as premature and multiple pregnancies, birthweight, and parents’ age. It occurs more commonly in boys than in girls.^6^, ^10^, ^11^ Currently, cranioplasty is the only treatment option available for craniosynostosis, but this surgical procedure is accompanied by an increased morbidity. Hence, there is an urgent need for developing new therapeutic modalities, such as pharmacological agents, that can delay or prevent premature suture fusion and in this way complement or even substitute surgery.

The genetic background and the underlying molecular mechanisms of the non‐syndromic subtype remain largely unknown. Recent reports have implicated mutations in specific genes, including *fibroblast growth factor receptors* (*FGFRs*), *transcription factor 12* (*TCF12*), *ETS2 repressor factor* (*ERF*), *TWIST*, *homeobox protein aristaless‐like* (*4ALX4*), *runt‐related transcription factor 2* (*RUNX2*) and *homeobox protein MSX‐2* (*MSX2*), whose products are components of cross‐talking signalling pathways.^2^, ^9^ The causative role of the mechanical microenvironment in the non‐syndromic form has been emphasized by recent studies that relate abnormal mechanical cues to the pathogenesis of craniosynostosis. Increased intrauterine forces developing due to multiple births and low pelvic station have been associated with non‐syndromic craniosynostosis.^12^, ^13^, ^14^ In addition, masticatory forces in osteopetrotic mice seem to induce premature fusion of the sagittal sutures, while soft diets administered to rats lead to premature fusion of the internasal suture.^12^, ^14^ Understanding the mechanobiology of craniosynostosis in detail will offer new insights into this disease and potentially lead to the development of novel targeted therapies.

Polycystins (PCs), a distinct family of transmembrane proteins comprising polycystin‐1 (PC1; encoded by *PKD1*) and polycystin‐2 (PC2; encoded by *PKD2*), have been shown to act as important regulators of mechanosensing, forming networks with other mechanosensitive partners and delivering mechanical signals within the cytoplasm that trigger biochemical responses.^15^ They are localized at the primary cilia, plasma membrane and endoplasmic reticulum. PC1 functions as an atypical G protein–coupled receptor with a long extracellular N‐terminal domain that senses mechanical signals. The intracellular PC1 C‐terminal tail interacts and activates several signal transduction pathways, including the Wnt, the activator protein‐1 (AP‐1), the mammalian target of rapamycin (mTOR), the Janus kinase (JAK)‐signal transducer and activator of transcription (STAT), and the calcineurin‐nuclear factor of activated T cells (NFAT) axes.^15^, ^16^, ^17^, ^18^ PC2 functions as a Ca^2+^‐permeable channel. PCs are expressed in several human tissues, including kidneys, blood vessels, pancreas, liver and bones. They are implicated in renal flow sensing, vascular pressure and flow mechanosensation, blood‐brain barrier mechanical injury, skeletal development and osteoblast differentiation, and lately cancer.^19^, ^20^, ^21^, ^22^, ^23^, ^24^, ^25^, ^26^, ^27^

In osteoblastic cells, mechanical cues sensed and transduced by PC1 ignite signalling pathways that affect proliferation and differentiation.^28^ Moreover, mechanical load up‐regulates the expression of the osteoblast differentiation‐specific *RUNX2* gene via potentiation of the PC1‐JAK2‐STAT3 and PC1‐calcineurin‐NFAT signalling axes culminating in osteoblastogenesis and ultimately bone formation.^29^, ^30^ Additionally, PC1‐deficient mice subjected to midpalatal suture expansion exhibited restricted growth effects at the skull base and in craniofacial sutures, and conditional deletion of PC1 in neural crest‐derived cells resulted in mutant mice displaying craniofacial deformities, such as abnormal skull shapes, alveolar bone loss, compressed temporomandibular joints, distorted incisors and fractured molar roots.^31^, ^32^

In the present study, we used IgPKD1, an antibody against the mechanosensing extracellular N‐terminal domain of PC1, to investigate the role of PC1 in craniosynostosis, specifically in cells obtained from suture tissue from patients with trigonocephaly and dolichocephaly. We examined whether PC1 modulates differentiation in human primary cranial suture cells by focusing on its effect on RUNX2 and the gene expression levels of *osteocalcin*, an osteoblast‐specific gene. This work demonstrates for the first time that PC1 regulates RUNX2 activation and *osteocalcin* gene expression, through an extracellular signal–regulated kinase (ERK)–dependent manner, in a human craniosynostosis cell model. Therefore, our in vitro findings provide further insight into the mechanobiology of craniosynostosis, highlighting PC1 as an important player in this disease.

## 2 MATERIALS AND METHODS

### 2.1 Tissue specimens

Seventeen suture bone fragments of non‐syndromic craniosynostosis patients (8 with trigonocephaly, 9 with dolichocephaly—median age 6 years, 13 males and 4 females) were collected in RNA later and formalin solution at the Neurosurgery Department of ‘Aghia Sofia’ Children's Hospital, Athens, Greece. Written informed consent from parents or guardians of children with craniosynostosis was obtained along with recording of clinicopathological characteristics (Apgar score, birthweight, premature birth, multiple or single pregnancy and the kind of birth; data not shown). The study was approved by the Ethics Committee of the National and Kapodistrian University of Athens and ‘Aghia Sofia’ Children's Hospital.

### 2.2 Antibodies and reagents

The following primary antibodies were employed for Western blot analysis: actin (MAB1501, Millipore), polycystin‐1 CT2741 (kindly provided by the Baltimore Polycystic Kidney Disease Research and Clinical Core Center, P30 DK090868) and phosphorylated RUNX2 (p‐RUNX2; AF7379, Affinity Biosciences, USA). The following secondary antibodies were used: goat anti‐mouse IgG HRP‐conjugate (AP124P, Millipore) and goat anti‐rabbit IgG HRP‐conjugate (AP132P, Millipore). The IgPKD1 antibody was a kind gift from Dr O. Ibraghimov‐Beskrovnaya and Herve Husson (Genzyme Co., Boston, MA).^33^, ^34^ Cells were treated with 50 μmole/l of a MEK inhibitor (PD98059, Sigma‐Aldrich, Germany).

### 2.3 Cell culture

Human periodontal ligament (PDL) fibroblasts were obtained from explant cultures of PDL tissues as detailed previously.^35^, ^36^ The cells were maintained in Dulbecco's modified Eagle's medium (DMEM) supplemented with 10% foetal bovine serum (FBS); all experiments were carried out with cells from third to sixth passage after being checked for their osteoblastic characteristics.

Explants of suture tissue from five patients with craniosynostosis (three with trigonocephaly and two with dolichocephaly) were obtained by collagenase digestion, and cranial suture cells were cultured according to previously described methods.^37^ In brief, the human suture tissue samples were dissected and minced into 1‐mm bone fragments and incubated in 0.25% collagenase for 2 hours at 37°C. Samples were then centrifuged, and the supernatant was removed. Pellets were extensively washed with phosphate‐buffered saline (PBS) and plated at 5 bone fragments per well in both 6‐well and 12‐well plates. Cells were cultured in minimal medium in a humidified atmosphere containing 5% CO_2_ at 37°C. Minimal medium consisted of aMEM (Gibco, Thermo Fisher Scientific, Germany), low glucose, supplemented with L‐glutamine, 10% FBS (Gibco, Thermo Fisher Scientific, Germany) and 1% antibiotics (penicillin 100 IU/ml, streptomycin 100 μg/ml) (Gibco, Thermo Fisher Scientific, Germany). Upon confluency, cells were plated in T25 flasks and labelled P1 (passage 1). Medium was changed every 2 days. Cells were passaged to P4 (passage 4) to obtain a sufficient amount of cells. All experiments were carried out with cells from P1 to P4 after being checked for their osteoblastic characteristics. For Western blotting and real‐time RT‐PCR, cells were incubated with IgPKD1 (dilution 1:50) for 3 hours and with MEK inhibitor (50 μmol/L) for 1 hour, followed by collection and analysis of extracts at different time‐points (6, 12 and 24 hours for Western blotting; 12 and 24 hours for real‐time RT‐PCR).

### 2.4 Western blot analysis

Protein extracts were resolved by electrophoresis in SDS‐polyacrylamide gels with varying densities (6% for PC1; 10% for p‐RUNX2) and transferred onto a nitrocellulose membrane (Porablot NCP, Macherey‐Nagel, Duren, Germany). Membranes were incubated overnight at 4°C with the primary antibodies [dilutions were 1:250 for antibodies against PC1 and 1:1000 for antibodies against p‐RUNX2 and actin in PBS‐0.1% Tween 20® detergent (PBST) containing 1% non‐fat milk]. Detection of the immunoreactive bands was performed with the LumiSensor Chemiluminescent HRP Substrate kit (GenScript). Relative protein amounts were evaluated by densitometric analysis using ImageJ software and normalized to the corresponding actin levels.

### 2.5 Reverse transcription (RT)‐PCR and semi‐quantitative PCR

Total RNA was extracted from cultured cells using RNeasy Mini Kit (Qiagen, Hilden, Germany) according to the manufacturer's instructions. PrimeScript RT reagent kit‐Perfect Real Time (Takara Bio) for RT‐PCR was used for cDNA synthesis according to the manufacturer's protocol. For semi‐quantitative PCR, the produced cDNA was amplified with specific primer pairs for the PC1‐encoding *PKD1* gene (annealing 58°C; forward: CGCCGCTTCACTAGCTTCGAC, reverse: ACGCTCCAGAGGGAGTCCAC) (35 cycles) as well as with *actin* gene specific primer pairs (28 cycles) using KAPA2G Fast Multiplex PCR Kit (KAPA Biosystems). PCR‐amplified fragments were analysed after their separation in agarose gels.

### 2.6 Real‐time RT‐PCR

Expression of *osteocalcin* and *gapdh* genes was determined by real‐time RT‐PCR using the iCycler MyiQ detection system (Bio‐Rad Laboratories), SYBRGreen (Roche), and specific primers for *osteocalcin* and *gapdh*. One microlitre of cDNA was amplified as a template in a 25‐μL reaction mixture containing 12.5 μL 2xSYBRGreen PCR Master Mix (Roche), 2.5 μL of primers and 9 μL deionized water. The mixture was heated initially at 95°C for 5 minutes followed by 40 cycles with denaturation at 95°C for 10 seconds and combined annealing/extension at 60°C for 30 seconds. Data were analysed by the comparative threshold cycle method.

### 2.7 Statistical analysis

Statistical analyses were conducted with the SPSS 23.0, Prism 6 and Microsoft Excel software packages. All experiments were performed at least three times, and representative results of one experiment are shown. The data are presented as mean ± SD for the number of experiments indicated and analysed by Student's *t* test. All statistical tests were two‐sided, and the value of *P* < .05 was regarded as statistically significant (*), with **P < .01, ***P < .001.

## 3 RESULTS

### 3.1 Detection of PC1 in cranial suture cells of trigonocephaly and dolichocephaly patients

**FIGURE 1:**
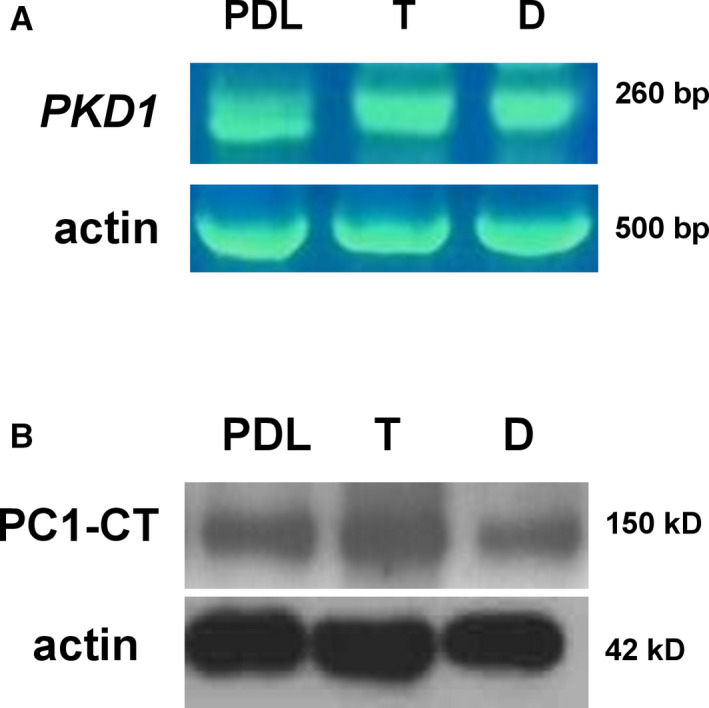
PC1 mRNA and protein expression levels in human primary cranial suture cells of trigonocephaly (T) and dolichocephaly (D). A, mRNA expression of *PKD1* gene in T and D cranial suture cells in comparison with the mRNA expression of *PKD1* gene in PDL cells (positive control). B, Protein expression of PC1 C‐terminal tail (PC1‐CT) in T and D cranial suture cells and PDL cells (positive control)

Until now, polycystins have been linked to craniosynostosis only in mouse models.^32^ Therefore, we firstly sought to determine the mRNA expression of the *PKD1* gene in primary cranial suture cells of trigonocephaly and dolichocephaly patients via RT‐PCR (Figure 1A). In addition, protein expression of the PC1 C‐terminal tail was evaluated in the same cells by Western immunoblotting (Figure 1B). For both RT‐PCR and Western immunoblot analysis, we used human periodontal ligament (PDL) cells, which were previously demonstrated to consistently express PC1, as a positive control.^29^ The results showed that PC1 is expressed in primary cranial suture cells of trigonocephaly and dolichocephaly patients both at the mRNA and protein level.

### 3.2 PC1 modulation triggers activation of RUNX2 through an ERK‐dependent manner in craniosynostosis

**FIGURE 2:**
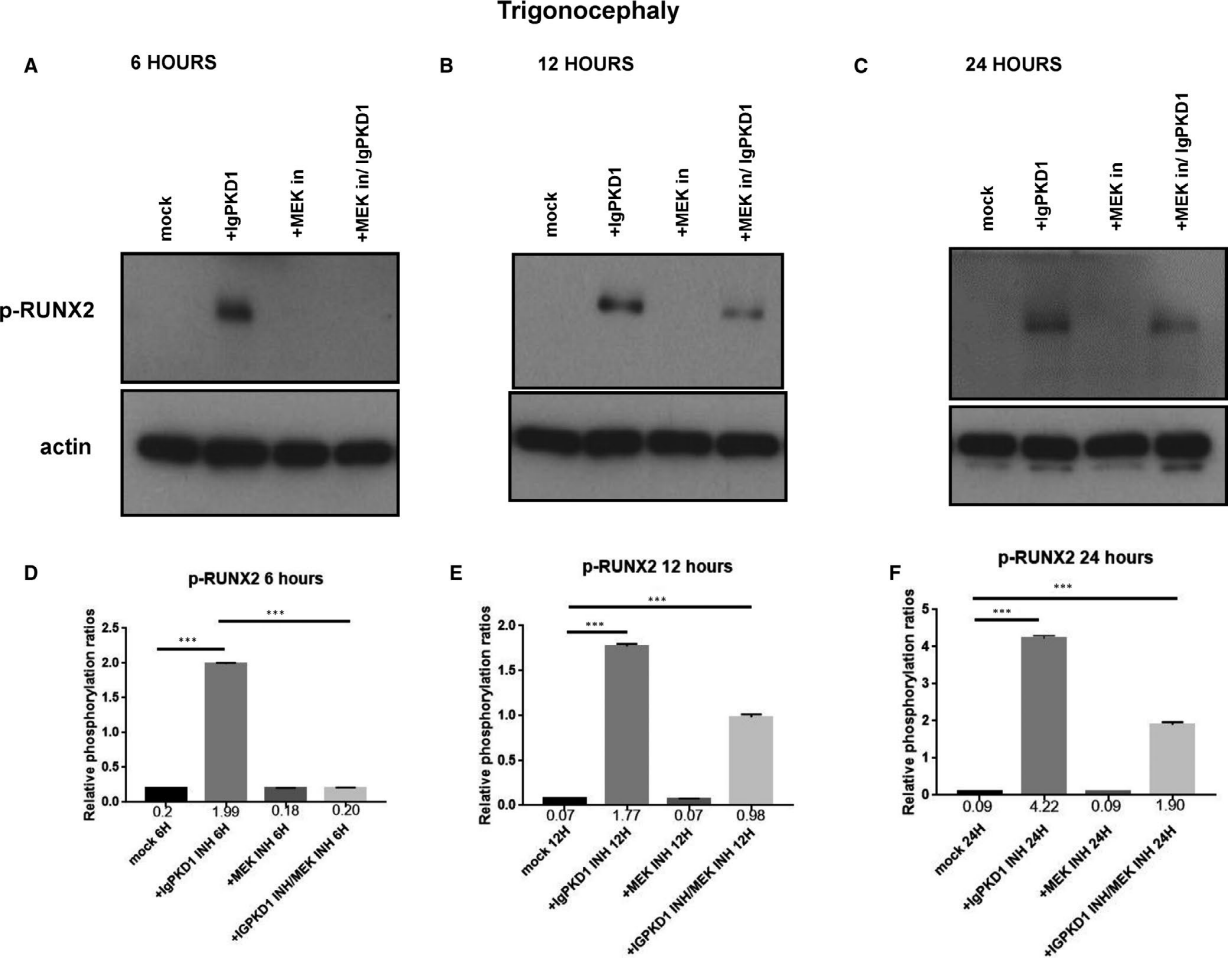
Effect of IgPKD1, MEK inhibitor and dual IgPKD1/MEK inhibitor treatment on phosphorylated RUNX2 (p‐RUNX2) in trigonocephaly cranial suture cells. A–C, Western blot analysis showing the effect of IgPKD1, MEK inhibitor and dual IgPKD1/MEK inhibitor treatment on the phosphorylation status of RUNX2 in trigonocephaly cells at 6, 12 and 24 h. D–F, Quantitative data showing the effect of IgPKD1, MEK inhibitor and dual IgPKD1/MEK inhibitor treatment on the phosphorylation status of RUNX2 in trigonocephaly cells at 6, 12 and 24 h. Bars represent means ± SD. ***P < .001

We carried on to explore the potential functional role of PC1 in our human craniosynostosis cell model. *RUNX2* is a strong candidate gene in craniosynostosis. Copy number variations have been found in cranial suture samples, and RUNX2 has been correlated with the occurrence of syndromic and non‐syndromic craniosynostosis.^2^ Furthermore, PC1 has been demonstrated to up‐regulate *RUNX2* gene expression in human primary osteoblast‐like cells.^29^, ^30^ To investigate whether an association between PC1 and RUNX2 exists in craniosynostosis, we sought to modulate PC1 via an antibody which binds to the mechanosensing extracellular N‐terminal domain of the protein, namely, IgPKD1, and assess its effect on the activation (phosphorylation) status of RUNX2 in trigonocephaly and dolichocephaly cranial suture cells. Specifically, we incubated trigonocephaly and dolichocephaly cranial suture cells with IgPKD1 for 3 hours and then harvested cells at different time‐points (6, 12, 24 h) for analysis. A MEK inhibitor (1 hour incubation) was also used in order to investigate if any effect of PC1 on RUNX2 activation is mediated through the ERK pathway, as ERK has been shown to phosphorylate and hence potentiate RUNX2 in mechanically stimulated osteoblastic cells.^36^, ^38^ In both trigonocephaly and dolichocephaly cranial suture cells, we observed that after IgPKD1 treatment, phosphorylated RUNX2 (p‐RUNX2) was significantly increased at all time‐points. This positive effect of PC1 on RUNX2 activation in trigonocephaly cells was more prominent at 6 and 24 h, whereas in dolichocephaly cells, it was more prominent at 6 and 12 h. Furthermore, after treatment with both IgPKD1 and the MEK inhibitor, p‐RUNX2 levels were significantly decreased in dolichocephaly cells at 6 and 24 hours compared to IgPKD1 treatment alone, while in trigonocephaly cells, p‐RUNX2 levels were negligible at 6 hours (Figures 2 and3). These data further support the findings from previous studies that RUNX2 is implicated in craniosynostosis and suggest that PC1 regulates RUNX2 phosphorylation in trigonocephaly and dolichocephaly through a mechanism that involves ERK activation.

**FIGURE 3:**
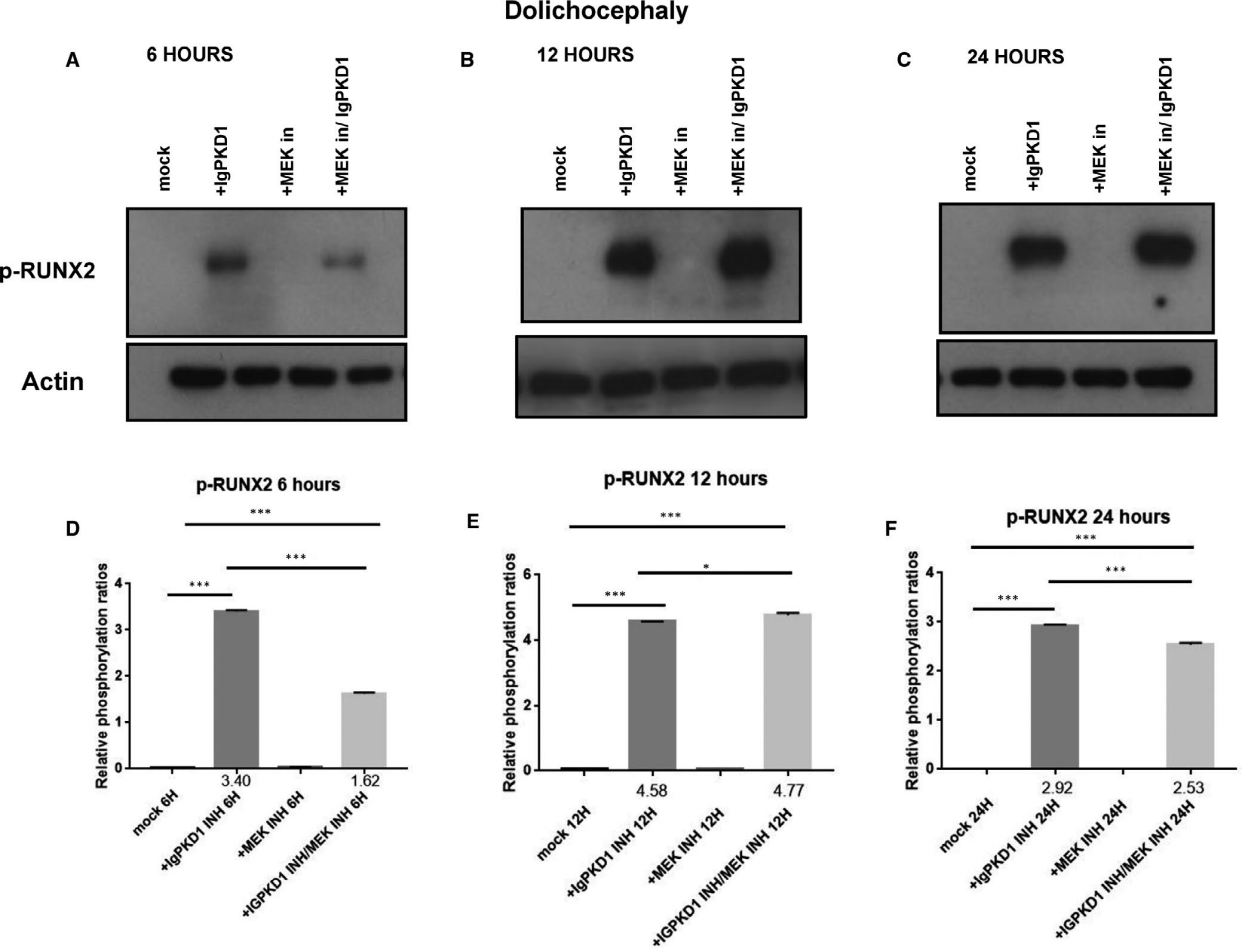
Effect of IgPKD1, MEK inhibitor and dual IgPKD1/MEK inhibitor treatment on phosphorylated RUNX2 (p‐RUNX2) in dolichocephaly cranial suture cells. A–C, Western blot analysis showing the effect of IgPKD1, MEK inhibitor and dual IgPKD1/MEK inhibitor treatment on the phosphorylation status of RUNX2 in dolichocephaly cells at 6, 12 and 24 h. D–F, Quantitative data showing the effect of IgPKD1, MEK inhibitor and dual IgPKD1/MEK inhibitor treatment on the phosphorylation status of RUNX2 in dolichocephaly cells at 6, 12 and 24 h. Bars represent means ± SD. *P < .05, ***P < .001

### 3.3 PC1 modulation induces up‐regulation of *osteocalcin* gene expression through an ERK‐dependent manner in craniosynostosis

**FIGURE 4:**
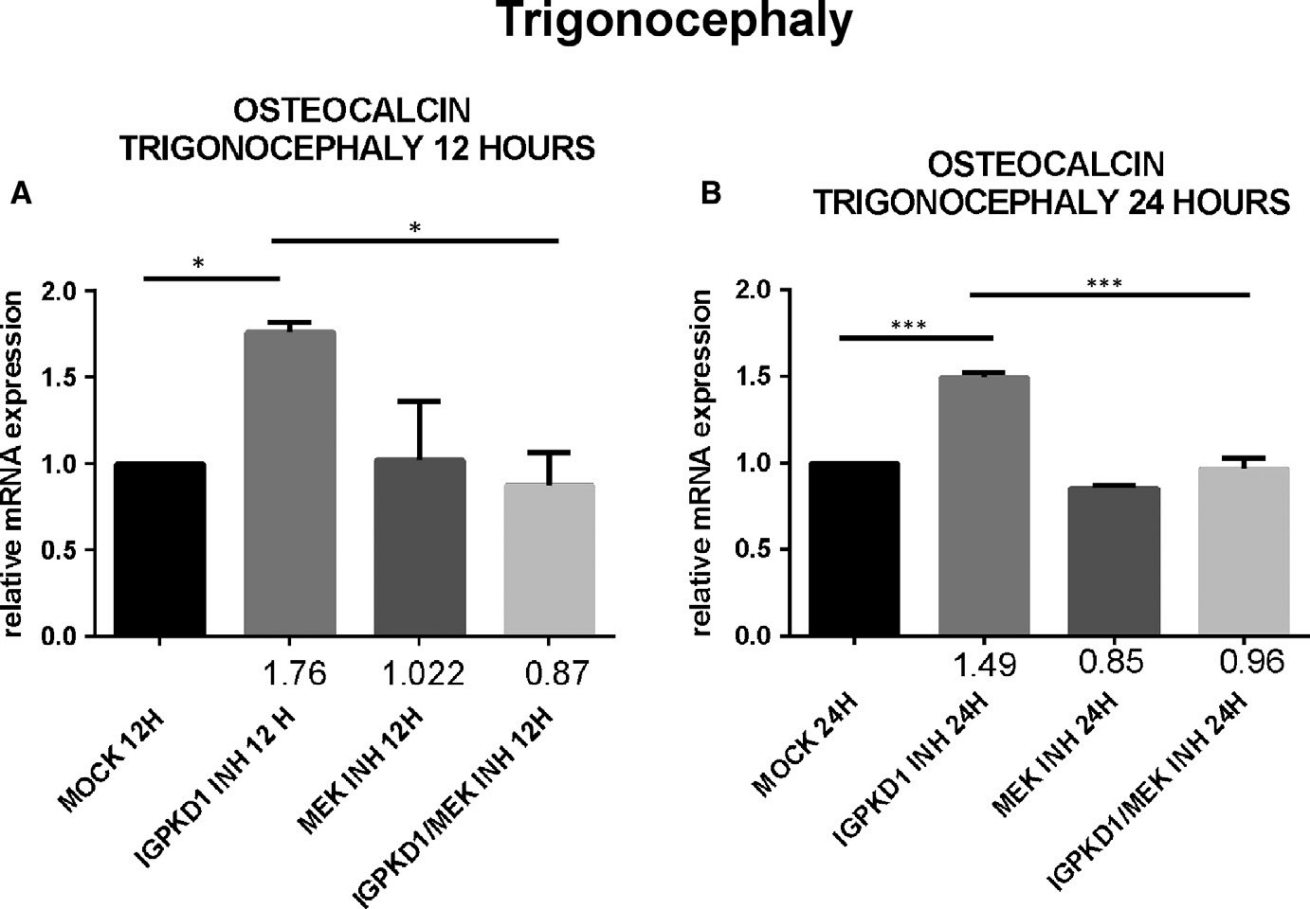
Expression of osteogenesis‐related biomarker osteocalcin following IgPKD1, MEK inhibitor and dual IgPKD1/MEK inhibitor treatment in trigonocephaly cranial suture cells. A, B, Quantitative data showing the mRNA expression of *osteocalcin* after IgPKD1, MEK inhibitor and dual IgPKD1/MEK inhibitor treatment of trigonocephaly cells compared to mock cells at 12 and 24 h. The results have been normalized to the housekeeping gene *gapdh*. *P < .05, ***P < .001

Lastly, since RUNX2 acts as a transcription factor that induces the expression of several osteoblastic genes, we explored if the previously detected PC1‐dependent RUNX2 activation also leads to up‐regulation of the expression of osteoblast‐specific genes, specifically *osteocalcin*. Cells were treated with either IgPKD1 or the MEK inhibitor, as well as their combination, and *osteocalcin* gene expression was evaluated at 12 and 24 h. Our data revealed that *osteocalcin* expression levels were significantly up‐regulated at 12 and 24 hours in trigonocephaly cells, while in dolichocephaly cells, *osteocalcin* gene expression was significantly augmented only at 24 hours after treatment with IgPKD1. When trigonocephaly cells were treated with IgPKD1 and the MEK inhibitor, *osteocalcin* expression levels were decreased at both time‐points compared to IgPKD1 treatment alone, whereas dual treatment in dolichocephaly cells did not exhibit any effect on *osteocalcin* gene expression compared to IgPKD1‐treated cells (Figures 4 and5). These findings indicate that PC1 affects the differentiation of primary cranial suture cells in both trigonocephaly and dolichocephaly by modulating the expression of the osteoblastic gene *osteocalcin*. The above results also suggest that PC1 controls the expression of *osteocalcin* in trigonocephaly cells via ERK signalling.

**FIGURE 5:**
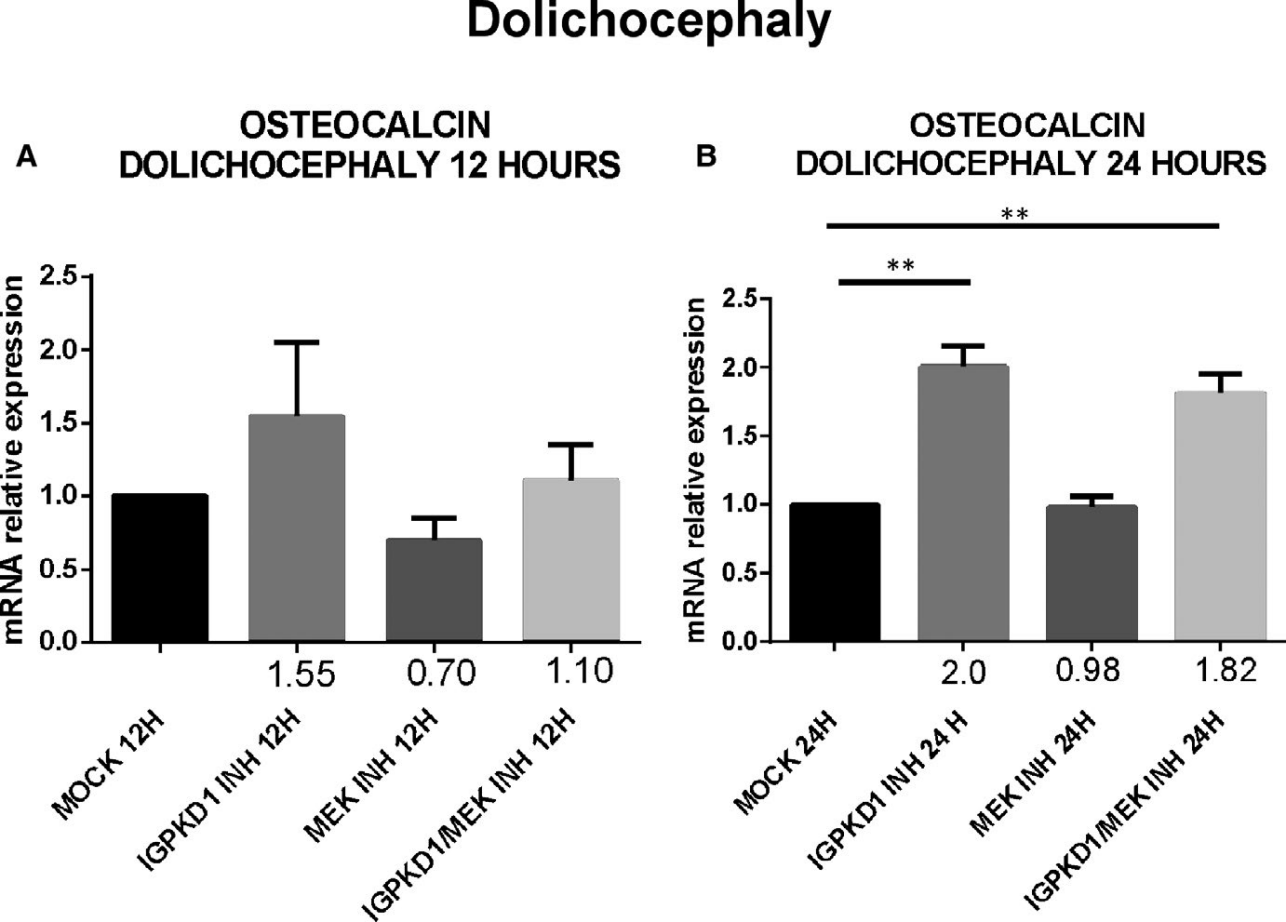
Expression of osteogenesis‐related biomarker osteocalcin following IgPKD1, MEK inhibitor and dual IgPKD1/MEK inhibitor treatment in dolichocephaly cranial suture cells. A, B, Quantitative data showing the mRNA expression of *osteocalcin* after IgPKD1, MEK inhibitor and dual IgPKD1/MEK inhibitor treatment of dolichocephaly cells compared to mock cells at 12 and 24 h. The results have been normalized to the housekeeping gene *gapdh*. **P < .01

## 4 DISCUSSION

Craniosynostosis is a pathological condition caused by premature suture closure that results in skull shape abnormalities associated with craniofacial growth changes, increased intracranial pressure, permanent brain injury, hearing loss and intellectual disability. Abnormal mechanical forces have recently been proposed to contribute to craniosynostosis, indicating that mechanosensory and mechanotransduction proteins may act as important pathogenic mediators.^12^, ^13^, ^39^

Previous studies in mouse models have demonstrated the implication of mechanosensory polycystins in cranial development.^31^, ^32^ In the present study, we investigated for the first time the presence and function of PC1 in cranial suture cells derived from human craniosynostotic tissues. We chose to perform our study in two common types of craniosynostosis, namely, trigonocephaly and dolichocephaly, to also evaluate if any differences exist between these two types in terms of molecular mechanisms. We focused on PC1 because of its main role in mechanosensation, cell‐to‐cell adhesion, cell‐matrix interactions, and the ability of its C‐terminal tail to interact with a plethora of intracellular signalling pathways.^15^, ^40^ In our primary cranial suture cells, we detected both mRNA and protein expression of PC1 in trigonocephaly and dolichocephaly.

Physiologically, mesenchymal osteoprogenitor cells in the ossification locus differentiate into osteoblasts which then produce organic osteoid matrix and initiate bone mineralization.^41^, ^42^, ^43^, ^44^ The outcome of this well‐orchestrated process is the formation of the bones of the cranial vault. As a result, we hypothesized that dysregulated osteoblast differentiation may lead to abnormal bone growth in cranial sutures giving rise to the pathological condition of craniosynostosis. Many genes involved in osteoblastic differentiation have been implicated in craniosynostosis, including *RUNX2*, *Indian hedgehog*, *Twist1*, *fibroblast growth factors* (*FGFs*) and *FGFRs*.^45^ Thus, following the detection of PC1 in our human craniosynostosis cell model, we proceeded to explore the functional role of PC1 in the cellular process of differentiation by focusing on its effect on the activation of RUNX2. Previous studies demonstrated that early onset of *RUNX2* expression leads to craniosynostosis.^46^ RUNX2 is the earliest transcription factor essential for osteoblast differentiation and bone formation.^47^, ^48^, ^49^ For our experiments, we used an antibody (IgPKD1) that binds to the extracellular N‐terminal domain of PC1 and blocks its mechanosensing ability in human osteoblastic cells.^29^, ^30^ A MEK inhibitor was also used to investigate if any effect of PC1 on RUNX2 activation was ERK mediated, since ERK is associated with potentiation (via phosphorylation) of RUNX2 in mechanically triggered osteoblastic cells.^36^, ^38^ Treatment of cranial suture cells with IgPKD1 for 6, 12 and 24 hours was found to significantly increase p‐RUNX2 levels in both trigonocephaly and dolichocephaly at all time‐points. Interestingly, the combination of IgPKD1 and MEK inhibitor resulted in complete reversal of RUNX2 activation at 6 hours in trigonocephaly cranial suture cells. RUNX2 phosphorylation was present at 12 and 24 hours in trigonocephaly cells and at all time‐points in dolichocephaly cells when both IgPKD1 and MEK inhibitor were used. However, it should be noted that in dolichocephaly cells, p‐RUNX2 was significantly decreased at 6 and 24 hours after combined IgPKD1/MEK inhibitor treatment compared to IgPKD1 treatment alone. All the above suggest that RUNX2 activation is vigorously regulated by PC1 in trigonocephaly and dolichocephaly cells and that ERK signalling may mediate this effect. These findings are in concert with previous data from our group which show that PC1 and ERK are linked to RUNX2 in human osteoblastic cells, and suggest that the association of PC1 with RUNX2 is probably a conserved molecular mechanism used by osteoblasts to control their differentiation.^29^, ^30^, ^36^

Regarding ERK, previous studies have highlighted its important role in craniosynostosis. For example, Lee et al showed that TGF‐β2 perturbation affects ERK signalling and results in craniosynostosis.^50^ Also, Miraoui et al demonstrated that ERK activation is involved in the osteogenic differentiation of mesenchymal stem cells carrying the S252W mutation in *fibroblast growth factor receptor 2* (*FGFR2*) associated with Apert syndrome, a rare congenital disorder characterized by craniosynostosis.^51^ Furthermore, craniosynostosis was significantly inhibited in a mouse model after treatment with a MEK inhibitor.^52^ In a mouse model for Apert syndrome, treatment of cultured calvaria and femur with an ERK1/2 inhibitor resulted in partial alleviation of coronal suture fusion and femur growth retardation.^53^ In relation to PC1, ERK signalling is up‐regulated in *PKD1*‐deficient mice skeletal tissue which is also observed in the context of polycystic kidney disease (PKD).^32^ Besides further supporting the role of ERK in craniosynostosis, our findings pose that ERK acts as a downstream effector of PC1 to activate RUNX2 and subsequently induce osteoblast differentiation in cranial suture cells from trigonocephaly and dolichocephaly patients.

Lastly, our previous finding that PC1 regulates RUNX2 activation prompted us to examine the effect of PC1 on the expression of osteogenic markers which are transcriptional targets of RUNX2. Several proteins are highly expressed during the mineralization stage of bone development, including osteocalcin (OC), osteopontin (OPN), bone sialoprotein (BSP‐II) and bone alkaline phosphatase (BALP) and are frequently used as markers for this stage in osteoblast differentiation.^54^ Therefore, we decided to analyse the mRNA levels of osteocalcin at 12 and 24 hours in cranial suture cells under the presence of IgPKD1, a MEK inhibitor, and their combination. We observed that IgPKD1 treatment significantly up‐regulated the transcription of the *osteocalcin* gene in both trigonocephaly and dolichocephaly cells at all time‐points except for 12 hours in dolichocephaly cells. The combination of IgPKD1 and MEK inhibitor led to a significant decrease in osteocalcin mRNA levels only in trigonocephaly cells, indicating that in dolichocephaly cells osteocalcin mRNA has probably a different turnover rate compared to trigonocephaly cells and/or that the kinetics of RUNX2 phosphorylation between trigonocephaly and dolichocephaly cells are probably dissimilar. These data justify the role of PC1 as regulator of cranial suture cell differentiation in both trigonocephaly and dolichocephaly, as well as the association of PC1 with ERK in craniosynostosis, at least in trigonocephaly.

Intriguingly, our results can be interpreted in two ways depending on the actual mechanism of action of IgPKD1 on PC1 in our craniosynostosis cell model. On the one side, IgPKD1 may act as an inhibitory antibody blocking PC1 from sensing extracellular mechanical cues and ferrying them intracellularly to trigger downstream biochemical pathways. According to this scenario, our data suggest that PC1 has a protective role in craniosynostosis, negatively regulating ERK and RUNX2 activation and consequently cranial suture cell differentiation; by hindering this function via IgPKD1, other positive intracellular signals take over and instigate ERK signalling, RUNX2 activation and finally cranial suture cell differentiation which promotes premature suture fusion. On the other side, IgPKD1 may act as a stimulatory antibody and once bound to the extracellular N‐terminal domain of PC1 it induces conformational alterations that may allow the C‐terminal tail to physically interact with intracellular proteins close to the membrane or to be cleaved, thus interfering with downstream signalling pathways. In this scenario, our findings indicate that PC1 promotes craniosynostosis, positively regulating ERK, RUNX2 activation and, in turn, cranial suture cell differentiation. Whether PC1 acts to promote or inhibit craniosynostosis remains to be clarified.

Mechanotransduction is a vital signalling process involved in osteoblast regulation and, thus, in diseases affecting the bone.^55^, ^56^ The present study unveils the PC1‐ERK‐RUNX2 axis as a novel mechanotransduction cascade in craniosynostosis with regulatory role over osteoblast‐specific gene expression required to develop a mature osteoblast phenotype. Given that there is a lack of prior research on the subject of polycystins and craniosynostosis in humans, our work represents the first steps towards understanding the function of PC1 in the pathophysiology of craniosynostosis. As expected, our findings raised many questions. Future experiments should dissect the mechanisms through which PC1 modulates cranial suture cell differentiation, and particularly the molecular details of the interaction among PC1, ERK and RUNX2. Moreover, future studies on polycystins and craniosynostosis should explore whether polycystins are associated with any other signalling pathways related to osteogenesis. Such knowledge may advance our understanding of craniosynostosis biology and perhaps lead to the identification of new therapeutic targets.

## 5 ETHICS APPROVAL AND CONSENT TO PARTICIPATE

The study was approved by the Ethics Committee of the National and Kapodistrian University of Athens and ‘Aghia Sofia’ Children's Hospital. All parents or guardians of patients had signed the informed consent.

## CONFLICT OF INTEREST

The authors declare that they have no conflict of interest.

## AUTHOR CONTRIBUTIONS

**Maira A. Katsianou:** Data curation (Supporting); Investigation (Lead); Methodology (Lead); Software (Equal); Writing‐original draft (Supporting). **Kostas A. Papavassiliou:** Data curation (Equal); Formal analysis (Equal); Validation (Equal); Writing‐original draft (Lead); Writing‐review & editing (Supporting). **Ilianna Zoi:** Data curation (Supporting); Investigation (Supporting); Methodology (Supporting). **Antonios N. Gargalionis:** Formal analysis (Supporting); Methodology (Supporting); Software (Equal). **Dimitrios Panagopoulos:** Resources (Lead). **Marios S. Themistocleous:** Resources (Lead). **Christina Piperi:** Conceptualization (Equal); Data curation (Equal); Formal analysis (Equal); Project administration (Equal); Supervision (Equal); Validation (Equal); Writing‐review & editing (Equal). **Athanasios G. Papavassiliou:** Conceptualization (Equal); Data curation (Lead); Formal analysis (Lead); Supervision (Equal); Validation (Lead); Visualization (Lead); Writing‐review & editing (Lead). **Efthimia K. Basdra:** Conceptualization (Lead); Data curation (Lead); Formal analysis (Lead); Project administration (Lead); Validation (Equal); Visualization (Equal); Writing‐original draft (Supporting).

## Data Availability

The data that support the findings of this study are available from the corresponding authors upon request.
